# Emotional pathways to engagement in AI-enhanced english learning: the role of AI literacy and foreign language enjoyment

**DOI:** 10.3389/fpsyg.2026.1872841

**Published:** 2026-07-31

**Authors:** Dongxue Bao, Juan Du

**Affiliations:** School of Foreign Languages, Hulunbuir University, Hulunbuir, China

**Keywords:** AI literacy, AI-enhanced English learning, classroom engagement, emotional pathways, foreign language enjoyment

## Abstract

**Purpose:**

Learner engagement is central to effective EFL learning, yet limited research has examined how AI literacy contributes to students' engagement in AI-enhanced language classrooms. Drawing on the growing recognition of emotion in technology-mediated learning, this study investigates whether foreign language enjoyment (FLE) functions as an affective mechanism through which AI literacy influences foreign language classroom engagement (FCE).

**Design:**

The study was conducted with 269 undergraduate students enrolled in AI-enhanced English courses at a Chinese university. This study integrated two complementary methodological approaches. First, structural equation modeling (SEM) was employed to explore the mediating role of FLE in the relationship between AI literacy (AIL) and classroom engagement. Second, latent profile analysis (LPA) was utilized to categorize students into homogeneous subgroups based on their FLE levels.

**Findings:**

SEM results showed that AI literacy significantly predicted foreign language enjoyment, which in turn positively predicted classroom engagement. The findings supported the mediating role of FLE in the relationship between AI literacy and classroom engagement. LPA identified two distinct learner profiles: a High-FLE group and a Low-FLE group. Learners in the High-FLE profile reported significantly higher levels of positive affect and classroom engagement, whereas those in the Low-FLE profile demonstrated lower enjoyment and weaker participation in AI-assisted learning activities.

**Value:**

This study advances research on AI-enhanced language learning by conceptualizing foreign language enjoyment as a key affective pathway linking AI literacy to classroom engagement. It reveals both the emotional mechanisms and learner heterogeneity underlying engagement in AI-integrated EFL classrooms. The findings offer pedagogical insights for designing emotionally responsive and pedagogically innovative AI-supported language learning environments.

## 1 Introduction

The rapid integration of artificial intelligence (AI) into tertiary language classrooms is reshaping how English is taught and experienced (Yuan et al., 2024). AI literacy is essential for students to understand, evaluate, and effectively use AI-driven tools introduced by instructors.This capacity significantly influences their ability to engage with these technologies responsibly and poses positive effect on their overall learning experience in higher education (Xia, 2025). These changes raise a critical pedagogical question: what enables learners to meaningfully appropriate AI technologies for language learning rather than merely use them instrumentally?

However, much of the existing research conceptualizes AI literacy primarily as a technical or cognitive capacity, focusing on what learners can do with AI tools (Almatrafi et al., 2024). Less attention has been paid to how AI literacy reshapes learners' emotional experiences in the classroom and, in turn, their engagement with language learning activities (Xie et al., 2026). Although recent research has begun to explore learners' motivation and enjoyment in AI-supported language learning (e.g., Zhang et al., 2026), the mechanisms linking AI literacy, emotional experiences, and classroom engagement remain largely unexplored. As a result, the mechanisms through which AI literacy translates into sustained foreign language classroom engagement (FCE) remain insufficiently theorized.

This study was conducted in an EFL classroom specifically designed as an application of the multidimensional AI literacy model proposed by Tour et al. (2025). The instructional design embodied the framework's operational, contextual, critical, and creative domains within a language learning context. This study examines how AI literacy relates to classroom engagement in AI-enhanced English learning by conceptualizing foreign language enjoyment (FLE) as an emotional Pathway rather than a mere outcome of learning. Specifically, it investigates whether AI literacy predicts engagement indirectly through FLE and whether learners can be differentiated into distinct enjoyment profiles with different patterns of AI literacy and engagement. By combining a variable-centered approach (SEM) and a person-centered approach (LPA), the study provides both processual and typological evidence of how students' emotional experiences shape engagement in innovative AI-mediated language classrooms.

## 2 Literature review

### 2.1 Theoretical foundation

The present study examines how AI literacy shapes students' engagement in AI-enhanced English learning, with foreign language enjoyment (FLE) functioning as a mediator and forming distinct learner profiles. This conceptualization reflects a multilevel mechanism linking cognition, emotion, and behavior. To explain this pathway, the study draws primarily on the Control–Value Theory of Achievement Emotions (Pekrun, 2006; Davis et al., 1989). These two frameworks were selected because they address complementary aspects of AI-enhanced language learning. CVT explains the emotional mechanism through learners' control–value appraisals, whereas TAM explains the role of technology-related perceptions in AI acceptance and use. Together, they offer an integrated perspective on how AI literacy influences foreign language enjoyment and engagement.

The Control–Value Theory (CVT) postulates that learners' academic emotions arise from two core cognitive appraisals: perceived control over learning activities and subjective value assigned to these tasks (Pekrun, 2006). When students believe they can manage learning demands effectively and view the task as meaningful or beneficial, they are more likely to experience positive achievement emotions such as enjoyment. Conversely, appraisals characterized by low control or low value tend to trigger negative affective responses, including anxiety and frustration. These emotions subsequently influence cognitive processing, motivation, strategy use, and behavioral engagement (Pekrun and nd Perry, 2014).

Within the context of AI-enhanced English learning, AI literacy functions as a key antecedent of control and value appraisals. Learners with higher levels of AI literacy—who feel capable of navigating AI tools, interpreting outputs, and integrating them into learning—are more likely to perceive AI-mediated tasks as controllable and useful. These appraisals, according to CVT, naturally boost enjoyment, a central positive achievement emotion associated with increased curiosity, interest, and willingness to participate. Thus, FLE serves as a theoretically grounded mediator linking cognitive evaluations formed through AI literacy to observable engagement behaviors.

To better capture the technological characteristics of AI-enhanced language learning, the present study complements the Control**–**Value Theory (CVT) with the Technology Acceptance Model (TAM). TAM proposed that learners' acceptance of educational technologies is primarily influenced by perceived ease of use and perceived usefulness (Davis et al., 1989). In the present study, these technology-related perceptions are viewed as shaping the control and value appraisals described in CVT. Specifically, learners who perceive AI tools as easy to use are more likely to feel in control of AI-supported learning tasks, whereas those who perceive AI tools as useful are more likely to value these learning activities. According to CVT, these appraisals foster foreign language enjoyment, which subsequently promotes learners' behavioral, cognitive, and emotional engagement. This integration is consistent with recent educational technology research suggesting that technology acceptance frameworks and achievement emotion theories provide complementary explanations of learning processes in technology-enhanced environments (Wu, 2026; Hebibi et al., 2025). Accordingly, the present study proposes that AI literacy enhances learners' perceptions of AI tools, thereby strengthening their control and value appraisals and ultimately promoting foreign language enjoyment and engagement.

This theoretical integration also informs the interpretation of learner profiles identified through LPA. Learners with different levels of AI literacy are expected to differ in their perceptions of AI-supported learning, leading to variations in control**–**value appraisals and, consequently, different levels of foreign language enjoyment. Thus, the identified high- and low-enjoyment profiles represent theoretically meaningful differences in learners' emotional and cognitive adaptation to AI-enhanced language learning rather than merely statistical groupings.

In summary, TAM explains how learners perceive AI technologies, whereas CVT explains how these perceptions develop into control**–**value appraisals that generate foreign language enjoyment and engagement. Together, the two frameworks provide a sequential cognitive**–**emotional mechanism for understanding how AI literacy contributes to engagement in AI-enhanced language learning.

### 2.2 AI literacy and foreign language engagement

AI literacy has become an essential skill for students in digital learning. It encompasses not only the ability to understand, use, and evaluate AI tools critically, but also the disposition to engage creatively and ethically with AI technologies in learning contexts (Ng et al., 2021; Tour et al., 2025). Within the field of foreign language education, AI literacy enables learners to utilize intelligent technologies such as AI-based writing assistants, adaptive translation tools, and conversational agents to support their learning process, thereby fostering deeper engagement with language tasks (Lan et al., 2025).

Foreign language engagement (FCE) refers to learners' behavioral, emotional, and cognitive involvement in classroom activities (Fredricks et al., 2004). AI literacy can nourish such engagement in several ways. First, AI tools promote autonomy and personalization, allowing learners to control learning pace and difficulty (Banik et al., 2025), which stimulates sustained behavioral engagement; Second, the interactive and feedback-rich environment created by AI systems encourages positive emotional experiences and reduces language anxiety (Primordia et al., 2025), thereby strengthening emotional engagement. Third, AI-based platforms facilitate metacognitive awareness and self-regulation (Akram et al., 2025), which are essential components of cognitive engagement.

Most studies have examined AI integration from a technological or pedagogical innovation perspective rather than from the learner's psychological engagement perspective (Malik et al., 2025; Obidovna, 2024). Thus, further empirical exploration is necessary to clarify how AI literacy contributes to enhancing learners' engagement in language classrooms.

### 2.3 AI literacy and foreign language enjoyment

AI literacy significantly influences on both student engagement and their emotional experiences in language learning. In the context of AI-enhanced education, learners' ability to understand and interact effectively with AI technologies may foster positive affective responses such as enjoyment, curiosity, and confidence (Yoon and Ahn, 2025). When students possess sufficient AI literacy, they are more likely to perceive AI tools as supportive learning partners rather than sources of uncertainty or threat, which contributes to a more enjoyable and stress-reduced learning atmosphere (Kang et al., 2025).

Foreign Language Enjoyment (FLE) is characterized by a positive emotional state that emerges when learners engage with language learning activities they consider stimulating, rewarding, or socially supportive (Dewaele and MacIntyre, 2014). The development of FLE is fundamentally shaped by learners' appraisals of control and value during AI-mediated learning activities (Pekrun, 2006). When students feel capable of operating AI tools and interpreting AI-generated outputs, their perceived control increases, making learning tasks more manageable and less cognitively taxing. AI-driven feedback and adaptive learning support can further strengthen this sense of control by offering immediate, tailored guidance that helps students monitor and adjust their performance (Prakash et al., 2024). At the same time, AI-enhanced tasks often highlight the usefulness and relevance of learning activities, thereby enhancing learners' value appraisals. Intelligent tools that demonstrate clear benefits, such as improving efficiency, accuracy, or comprehension, can increase students' perceptions of task importance and meaningfulness (Leng, 2025). These heightened control and value appraisals jointly contribute to more frequent and intense experiences of enjoyment. Furthermore, AI-supported collaborative platforms can create shared learning spaces where peer discussion, co-construction, and collective problem-solving foster socially enriched forms of enjoyment (Peng and Cheng, 2023). Through these mechanisms, AI-mediated learning environments provide both cognitive and social conditions that are conducive to the emergence of FLE.

Recent studies have begun to document these positive emotional outcomes. However, most existing literature remains conceptual, and empirical validation of the link between AI literacy and FLE is still in scarcity. Understanding this relationship is vital, as enjoyment represents a key emotional driver of persistence and creativity in language learning (Dewaele and MacIntyre, 2014). By developing AI literacy, educators may indirectly promote more positive emotional engagement and wellbeing in AI-enhanced EFL contexts.

### 2.4 Foreign language enjoyment and engagement

Foreign Language Enjoyment (FLE), now widely viewed as a central affective factor, plays a growing role in sustaining learners' engagement within the language classroom (Dewaele and MacIntyre, 2014). From a Control–Value Theory perspective, FLE arises when AI-supported learning increases students' perceived control and task value through useful, easy-to-use tools and adaptive feedback (Dewaele and MacIntyre, 2014). When students experience enjoyment, they are more likely to invest cognitive effort, participate actively, and persist in challenging tasks, which collectively contribute to higher levels of Foreign Language Classroom Engagement (FCE) (Zeng, 2021). In language learning, this means that enjoyment not only motives attention and willingness to communicate but also encourages strategic risk-taking and social interaction (Aljasir, 2024). Empirical evidence supports that students with higher FLE demonstrate more behavioral, emotional, and cognitive engagement (Almutlaq, 2024; Zeng, 2021; Zhong, 2024).

Moreover, engagement can be seen as the behavioral manifestation of the internal motivational energy sparked by enjoyment (Peng and Cheng, 2023). When learners find joy in classroom interactions, they are more attentive, collaborative, and persistent in tasks, suggesting that FLE not only coexists with but actively drives FCE. In AI-enhanced learning environments, where intelligent tools provide immediate feedback and gamified learning experiences, such affective uplift may be even more pronounced (Kaur et al., 2025).

In summary, prior studies and theoretical work both view enjoyment as a key bridge to higher engagement. This relationship lays the conceptual foundation for examining FLE as a mediator in the predictive pathway from AI literacy to classroom engagement.

### 2.5 Foreign language enjoyment as a mediator between AI literacy and engagement

Although existing studies have separately examined the links between AI literacy, foreign language enjoyment (FLE), and engagement, little is known about how these constructs interact as a system. Building upon both Control–Value Theory (Pekrun, 2006), it is reasonable to believe that FLE serves as an emotional bridge connecting learners' AI-related capabilities with their behavioral and cognitive engagement in language learning.

AI literacy positively stimulates learners' perceived control and understanding of AI-enhanced learning environments, enabling them to feel more competent and capable in managing academic tasks. Such heightened control and value appraisals generate positive achievement emotions, including enjoyment, as proposed by Control–Value Theory (Pekrun, 2006). These positive experiences foster personal growth and resilience, translating into greater confidence and adaptability in academic contexts, thus promoting sustained engagement (Shi and Xu, 2025). In other words, students who feel more capable of utilizing AI tools (e.g., intelligent feedback, adaptive tasks, creative generation) are more likely to experience joy and curiosity in learning, which in turn stimulates greater effort and persistence.

Moreover, FLE captures the affective resonance between learners and their AI-mediated learning experiences. Positive interactions with intelligent systems, such as personalized feedback or gamified exercises, can generate enjoyment, reinforcing learners' motivation to participate and concentrate (Guo and Wang, 2025). This is consistent with a core premise of Control–Value Theory (Pekrun, 2006): positive emotions such as enjoyment emerge when learners perceive high control over tasks and attach substantial value to them, thereby fostering deeper engagement. In this sense, enjoyment functions not merely as an outcome of AI-supported learning but as a key emotional mechanism through which AI literacy translates into engagement.

Despite these theoretical connections, empirical validation of this mediating pathway remains limited. Most AI-enhanced learning studies have focused on cognitive or performance outcomes rather than affective mechanisms (Ambarita and Nurrahmatullah, 2024). Therefore, investigating FLE as a mediator between AI literacy and engagement can fill a critical gap, offering new insight into how emotional experiences underpin effective AI-mediated language learning.

### 2.6 Variable-centered and person-centered approaches

To achieve a more comprehensive understanding of learners' behavioral and emotional patterns, recent studies have increasingly advocated integrating variable-centered and person-centered approaches. The variable-centered approach (e.g., structural equation modeling and mediation analysis) is well-suited for examining directional relationships among constructs such as AI literacy, foreign language enjoyment (FLE), and engagement. It helps clarify how individual variables contribute to learners' emotional and behavioral outcomes in AI-enhanced contexts (Howard and Hoffman, 2018). However, this approach assumes population homogeneity and may overlook the fact that students differ substantially in their levels of affective experiences (Woo et al., 2024). In reality, learners have varying levels of technological competence and emotional responses. An exclusive focus on average effects risks overlooking the meaningful variation within the group.

To address this limitation, the present study integrates a person-centered approach, Latent Profile Analysis (LPA), to complement variable-centered modeling. LPA identifies subgroups of learners who share similar patterns across key constructs, allowing researchers to uncover the combinations of AI literacy, FLE, and engagement (Woo et al., 2024). This combined method not only verifies the mediating relationships through structural modeling but also captures the complex diversity of students' emotional and behavioral engagement in AI-supported language classrooms.

Recent research in applied linguistics and educational psychology has increasingly adopted LPA to explore individual differences in motivation, affect, and engagement (Feng et al., 2023; Li et al., 2023; Zhan and Zhong, 2025). For example, Li et al. (2023) demonstrated that person-centered approaches can reveal meaningful motivational differences among EFL learners and their associations with learning outcomes. More recently, Zhang et al. (2026) extended this line of inquiry to AI-supported language learning by adopting a person-centered approach to explore heterogeneous motivation and enjoyment patterns among Chinese university students in GenAI-assisted academic writing. However, neither study examined AI literacy as a key antecedent of learners' emotional experiences and classroom engagement, leaving this relationship insufficiently understood.

Despite these advances, few studies have applied LPA to investigate learners' affective and behavioral patterns in AI-enhanced EFL learning. Integrating variable- and person-centered approaches therefore enables a more comprehensive understanding of how AI literacy and FLE interact across different learner profiles—bridging the gap between group-level mechanisms and individual-level diversity.

### 2.7 The present study

To sum up, previous studies have highlighted the importance of AI literacy (AIL), foreign language enjoyment (FLE), and classroom engagement (FCE) in enhancing EFL learning outcomes. However, limited research has systematically examined the interrelationships among these constructs within a single model. Moreover, although previous studies have often treated FLE as a single-dimensional construct, the heterogeneity of learners' enjoyment experiences remains underexplored.

To address these gaps, the present study focuses on clarifying how AIL influences FCE through FLE as a mediator, using a variable-centered approach operationalized as structural equation modeling (SEM), alongside a person-centered perspective. In parallel, the person-centered analysis (i.e., LPA) focuses on identifying distinct subgroups of learners based on their levels and patterns of FLE.

Specifically, this study addresses three research questions:

RQ1: how does AI literacy (AIL) influence learners' foreign language enjoyment (FLE) and classroom engagement (FCE), and does FLE mediate this relationship?

RQ2: what are the distinct learner profiles based on their levels and patterns of foreign language enjoyment (FLE)?

RQ3: whether engagement (FCE) differs significantly across the identified FLE profiles remains a key question of this study?

**Figure 1:**
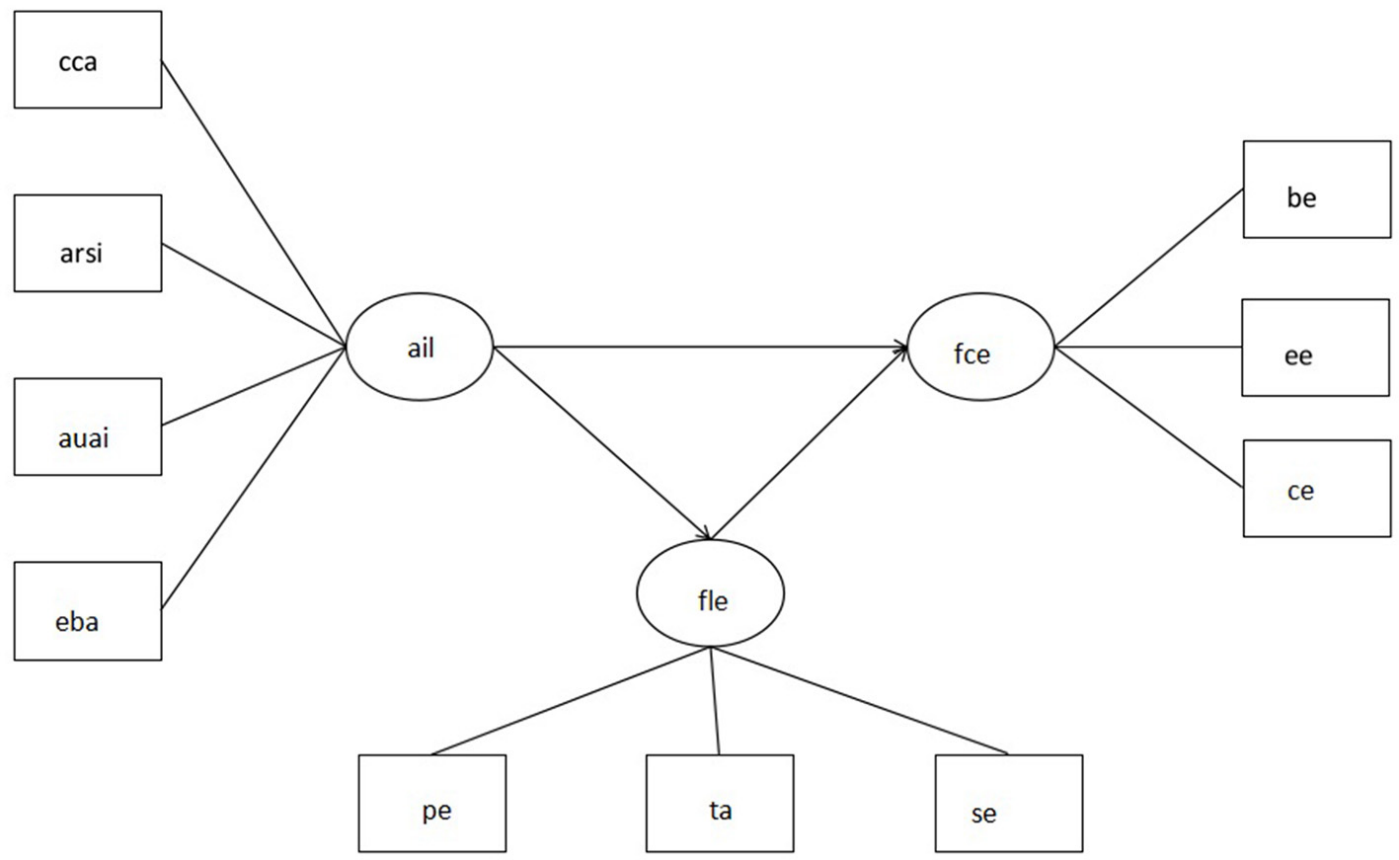
The hypothesized mediation model. AIL, AI literacy; FLE, foreign language enjoyment; FCE, foreign language classroom engagement. AIL dimensions: CCA, critical comprehension ability; ARAI, the ability to recognize the social impact of artificial intelligence; AUAI, the ability to use artificial intelligence technology; EBA, ethical behavior ability. FLE dimensions: PE, private enjoyment; SE, social enjoyment; TA, teacher appreciation. FCE dimensions: BE, behavioral engagement; CE, cognitive engagement; EE, emotional engagement.

A mediation model was constructed for the variable-centered analysis based on the literature review and theoretical framework. The model proposes that AI literacy is associated with foreign language enjoyment (FLE) and foreign language classroom engagement (FCE), with FLE serving as a mediator in the relationship between AI literacy and FCE. The proposed model is presented in Figure 1.

## 3 Methods

### 3.1 Participants

A total of 302 first-year non-English major students from a comprehensive university in China participated in this study. After data screening, 269 valid responses (89.1%) were retained for analysis. A Monte Carlo Power Analysis for Indirect Effects indicated that a minimum of 229 participants would be sufficient to detect the mediation effect, demonstrating that the final sample size was adequately powered. Among the participants, 162 (60.2%) were female and 107 (39.8%) were male; 134 (49.8%) majored in science and technology, while 135 (50.2%) were from social sciences and humanities.

All participants completed a 12-week AI-enhanced English course, consisting of one 90-min session per week. The course was designed based on the sociocultural AI literacy model proposed by Tour et al. (2025), which conceptualizes AI literacy as the development of operational, contextual, critical, and creative capabilities required for meaningful interaction with AI in language learning contexts. Rather than teaching AI literacy as a separate module, these capabilities were integrated into regular English learning activities to promote the simultaneous development of language competence and AI literacy.

Throughout the course, students engaged in AI-supported tasks embedded within routine classroom instruction. Typical activities included generating and refining prompts for language tasks, using generative AI to support reading, writing and translation, comparing AI-generated and human-produced texts, evaluating the accuracy and appropriateness of AI outputs, and reflecting on the ethical and responsible use of AI in academic contexts.

The questionnaire was administered anonymously on Wenjuanxing platform. Participation was voluntary, and informed consent was obtained from all respondents prior to data collection. Ethical approval for this study was granted by the Academic Ethics Committee of the School of Foreign Languages at the researcher's institution.

### 3.2 Instruments

#### 3.2.1 AI literacy (AIL)

Students' AI literacy was assessed using the Digital Literacy Scale developed by Hwang et al. (2023). The instrument was selected because it provides a validated multidimensional measure of AI literacy, assessing learners' Critical Comprehension Ability, Ability to Recognize the Social Impact of Artificial Intelligence, Ability to Use Artificial Intelligence Technology, and Ethical Behavior Ability. These dimensions broadly align with the operational, contextual, and critical domains of the AI literacy model proposed by Tour et al. (2025), while Ethical Behavior Ability complements Tour et al.'s emphasis on ethical dispositions in AI literacy practices. Although Hwang et al.'s scale does not include a separate creative dimension, it captures the core competencies targeted by the present intervention. Accordingly, Tour et al.'s model informed the instructional design, whereas Hwang et al.'s scale was used to assess students' AI literacy outcomes. Each item was rated on a five-point Likert scale (1 = strongly disagree, 5 = strongly agree). In the present study, Cronbach's alpha coefficients for the four subscales were 0.832, 0.865, 0.940, and 0.934, respectively.

#### 3.2.2 Foreign language classroom engagement (FCE)

Foreign language classroom engagement was measured using the 9-item Language Classroom Engagement Scale (LCES) developed and validated by (Liu et al. 2024). The scale comprises three subscales. All items were rated on a 5-point Likert scale (1 = strongly disagree, 5 = strongly agree). Regarding internal consistency, the three subscales demonstrated satisfactory reliability, with Cronbach's α values of 0.910, 0.921, and 0.896.

#### 3.2.3 Foreign language classroom enjoyment (FLE)

Foreign language enjoyment was assessed using the 9-item Short Form of the Foreign Language Enjoyment Scale developed and validated by Botes et al. (2021). The scale consists of three subscales. Each item was rated on a 5-point Likert scale ranging from 1 (strongly disagree) to 5 (strongly agree). The Cronbach's alpha values for the three subscales in this study were 0.888, 0.953, 0.925.

### 3.3 Analysis

To capture both the mediating mechanism and individual differences in emotional experiences in AI-enhanced EFL learning, this study adopted a mixed analytical design combining variable-centered and person-centered approaches. All data analyses were performed using SPSS 27.0 and Mplus 8.3. Descriptive and correlational analyses were first conducted in SPSS to examine the means, standard deviations, and interrelationships among AI literacy (AIL), foreign language classroom engagement (FCE), and foreign language enjoyment (FLE). Confirmatory factor analysis (CFA) was then implemented in Mplus 8.3 to validate the measurement models of the three constructs. After satisfactory model fit was achieved, structural equation modeling (SEM) was applied to test the hypothesized relationships among AIL, FCE, and FLE, with FLE serving as a mediating variable.

All CFA and SEM analyses employed the maximum likelihood estimation (MLE) method. Model fit was evaluated using multiple indices recommended by Kline (2015), including the chi-square to degrees of freedom ratio (χ^2^/df), root mean square error of approximation (RMSEA), comparative fit index (CFI), Tucker–Lewis index (TLI), and standardized root mean square residual (SRMR). Acceptable model fit was indicated by χ^2^/df < 5, RMSEA and SRMR < 0.08, and both CFI and TLI > 0.90. These standards have been broadly utilized in applied linguistics studies (Liu et al., 2025). The bias-corrected bootstrapping method (1,000 resamples, 95% confidence interval) was used to test the mediating effect of FLE between AIL and FCE.

Latent profile analysis (LPA) was subsequently conducted in Mplus 8.3 to identify distinct subgroups of learners based on their levels and patterns of foreign language enjoyment. A sequence of models with two to four profiles was tested to ascertain the optimal solution. Model selection was guided by several fit indices, including the Akaike Information Criterion (AIC), Bayesian Information Criterion (BIC), sample-size adjusted BIC (aBIC), the Lo–Mendell–Rubin Likelihood Ratio Test (LMR–LRT), the Bootstrap Likelihood Ratio Test (BLRT), and entropy. Lower AIC, BIC, and aBIC values indicated better model fit, while significant *p*-values (*p* < 0.05) of the LMR–LRT and BLRT suggested that the K-profile model fit better than the K−1 model (Asparouhov and Muthén, 2014). An entropy value above 0.70 was considered indicative of satisfactory classification accuracy. Following identification of the optimal profile solution, one-way ANOVAs were used to examine differences in behavioral, emotional, and cognitive engagement in relation to the identified FLE profiles.

## 4 Results

### 4.1 Descriptive and correlational analysis

Before testing the structural model, descriptive and correlational analyses were conducted to examine the distributions of the variables and the associations among the main constructs. As shown in Table 1, all skewness and kurtosis values fell within the acceptable range of ±3, indicating approximate univariate normality (Kline, 2015). Foreign language classroom engagement (FCE) was strongly and positively correlated with foreign language enjoyment (FLE) (*r* = 0.77, *p* < 0.01) and moderately correlated with AI literacy (AIL) (*r* = 0.57, *p* < 0.01). In addition, FLE showed a moderate positive correlation with AIL (*r* = 0.64, *p* < 0.01). These findings indicate that learners with higher levels of AI literacy tended to report greater foreign language enjoyment and classroom engagement. To assess the potential influence of common method bias, a single-factor confirmatory factor analysis (CFA) was conducted following the recommendations of Podsakoff et al. (2003). The one-factor model showed poor fit to the data (χ^2^ = 936.55, df = 35, CFI = 0.676, TLI = 0.584, RMSEA = 0.309, SRMR = 0.118), indicating that a single latent factor could not adequately account for the covariance among the observed variables. Therefore, common method bias was unlikely to pose a serious threat to the findings.

**Table 1:** Descriptive statistics and correlations.

Variables	1	2	3	M	SD	Skewness	Kurtosis
1. FCE	—			4.21	0.75	−0.49	−0.97
2. FLE	0.77^**^	—		4.37	0.64	−0.81	−0.47
3. AIL	0.57^**^	0.64^**^	—	3.96	0.64	0.04	−1.03

^**^p < 0.01. FCE, foreign language classroom engagement; FLE, foreign language enjoyment; AIL, AI literacy.

### 4.2 SEM result

Results of three confirmatory factor analyses (CFAs) for AIL, FLE, and FCE demonstrated generally acceptable measurement model fit. For AIL, the fit indices were χ^**2**^/df = 2.964, CFI = 0.928, TLI = 0.917, RMSEA = 0.052, and SRMR = 0.048. For FLE, χ^**2**^/df = 2.843, CFI = 0.935, TLI = 0.923, RMSEA = 0.050, and SRMR = 0.045. For FCE, χ^**2**^/df = 3.344, CFI = 0.978, TLI = 0.966, RMSEA = 0.093, and SRMR = 0.020. The structural equation model yielded the following fit indices: χ^**2**^/df = 6.01, CFI = 0.942, TLI = 0.919, RMSEA = 0.136, and SRMR = 0.041. Although the CFI, TLI, and SRMR indicated acceptable model fit, the χ^**2**^/df and RMSEA exceeded commonly recommended thresholds, suggesting that the overall structural model fit was not optimal. Therefore, the structural relationships should be interpreted with appropriate caution. Nevertheless, because multiple fit indices (CFI, TLI, and SRMR) reached acceptable levels and the model was theoretically grounded, the SEM results were retained for hypothesis testing. To examine potential multicollinearity, variance inflation factor (VIF) values were calculated using regression analysis for AIL and FLE as predictors of FCE. Both AIL and FLE showed a VIF value of 1.769, which was below the recommended threshold of 5, indicating that multicollinearity was not a serious concern.

AIL and FLE accounted for a substantial proportion of the variance in FCE (*R*^2^ = 0.794), indicating that the model explained nearly 80% of the variability in learners' classroom engagement. Shown as Table 2, the mediation model showed that the indirect effect of AIL on FCE through FLE was strong and significant (Estimate = 0.871, SE = 0.105, Z = 8.299, *p* < 0.001), with a 95% confidence interval of (0.677, 1.091), indicating that the mediation effect was robust. This large indirect effect suggests that AI literacy may nurture classroom engagement primarily by fostering learners' foreign language enjoyment. In contrast, the direct effect of AIL on FCE was negative and non-significant (Estimate = −0.090, SE = 0.100, *p* = 0.367), suggesting that AIL did not independently predict FCE once FLE was included in the model. The total effect of AIL on FCE remained significantly positive (Estimate = 0.781, SE = 0.081, *p* < 0.001), demonstrating that the influence of AIL on FCE operates primarily through FLE.

**Table 2:** Direct, indirect, and total effects of the mediation model.

Path	Estimate	Standardized effect (β)	SE	*Z*	95% CI Lower	95% CI Upper	*p*-value
AIL → FLE → FCE	0.871	0.661	0.105	8.299	0.677	1.091	< 0.001^***^
AIL → FCE	−0.090	−0.068	0.100	−0.902	−0.284	0.110	0.367
AIL → FCE (total effect)	0.781	0.592	0.081	9.643	0.628	0.941	< 0.001^***^

AIL, AI literacy; FLE, foreign language enjoyment; FCE, foreign language classroom engagement. CI based on 5000 bootstrap samples. ^***^p < 0.001.

**Figure 2:**
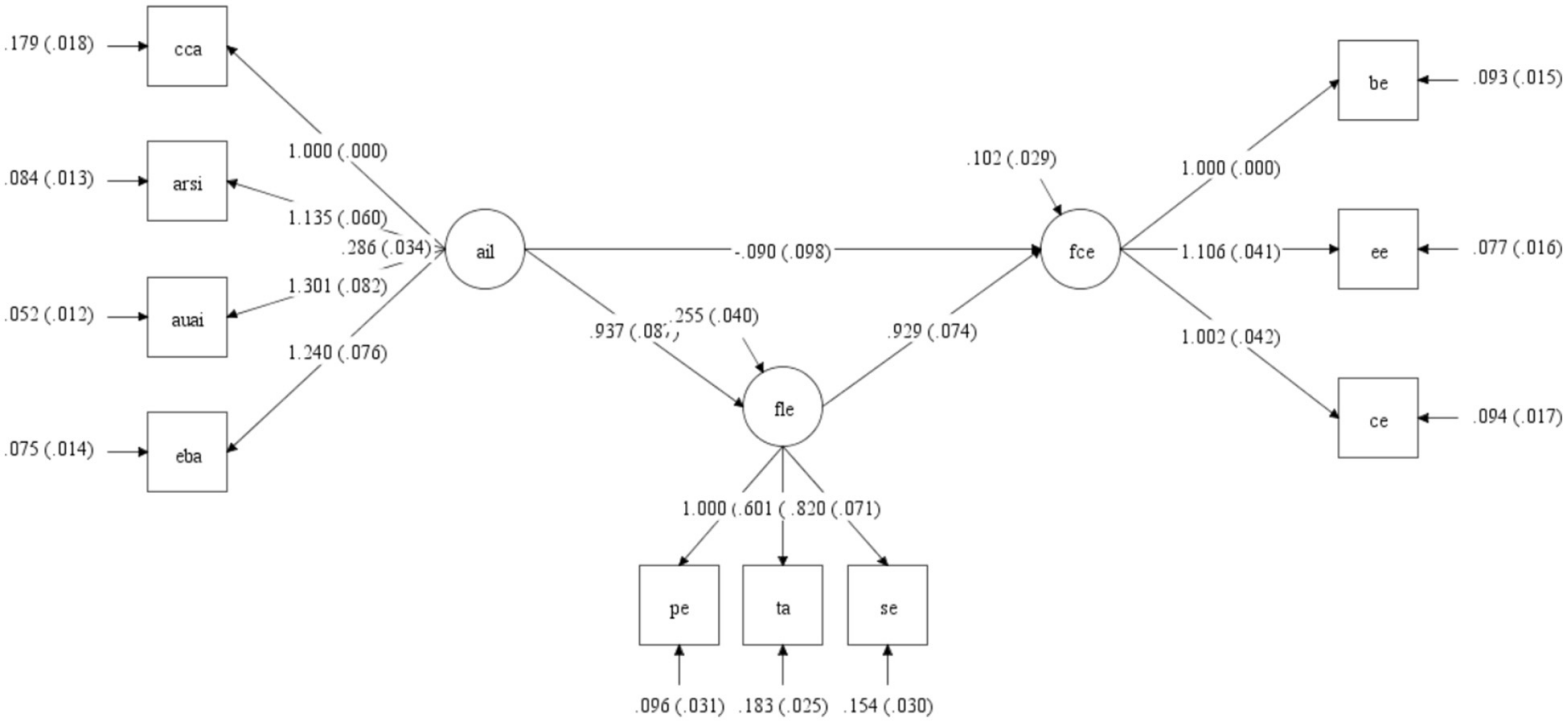
SEM results of the relationship among AIL, FLE and FCE. AIL, AI literacy; FLE, foreign language enjoyment; FCE, foreign language classroom engagement. AIL dimensions: CCA, critical comprehension ability; ARAI, The ability to recognize the social impact of artificial intelligence; AUAI, The ability to use artificial intelligence technology; EBA, ethical behavior ability. FLE dimensions: PE, private enjoyment; SE, social enjoyment; TA, teacher appreciation. FCE dimensions: BE, behavioral engagement; CE, cognitive engagement; EE, emotional engagement.

These findings provide strong support for the hypothesized full mediation, indicating that FLE is the mechanism through which AIL shapes students' foreign language classroom engagement. As illustrated in Figure 2, the hypothesized mediation model examined the structural relationships among AIL, FLE, and FCE.

### 4.3 LPA result

Latent profile analysis (LPA) was conducted to identify distinct subgroups of learners based on their Foreign Language Enjoyment (FLE). Table 3 presents the model fit indices for the two- to four-class solutions.

**Table 3:** Latent profile analysis.

Classes	AIC	BIC	aBIC	LMR-A *p* value	BLRT *p* value	Entropy	The smallest profile size (%)
2	3918.097	4018.749	3929.97	0.015	0	0.993	36.8
3	3001.38	3137.979	3017.494	0.824	0	1	7.06
4	2602.873	2775.419	2623.228	0.486	0	0.98	7.06

**Table 4:** Means of FLE scores and comparison across groups.

Group	*N*	Mean (SD)	SE	95% CI (Lower–Upper)	*F*	*p*	Partial η^2^
Low-FLE	99	3.68 (0.62)	0.06	3.56–3.81			
High-FLE	170	4.51 (0.65)	0.05	4.41–4.61	105.393	< 0.001	0.283
Total	269	4.21 (0.75)	0.05	4.12–4.30			

As shown in Table 3, the AIC, BIC, and adjusted BIC values decreased as the number of classes increased, suggesting incremental improvements in model fit. However, the Lo**–**Mendell**–**Rubin adjusted likelihood ratio test (LMR-A) was significant only for the two-class model (*p* = 0.015), but not for the three-class (*p* = 0.824) or four-class (*p* = 0.486) solutions, indicating that adding more profiles did not significantly improve model fit. Although the BLRT remained significant for the three- and four-profile solutions, the LMR-A test was not significant, indicating that additional profiles did not significantly improve model fit. Moreover, the three- and four-profile solutions produced a very small profile (7.06% of the sample) without yielding substantively distinct patterns beyond differences in overall FLE levels. Therefore, considering statistical evidence, class size, parsimony, and profile interpretability, the two-profile solution was retained. Therefore, the two-class solution was selected for its statistical adequacy, interpretability, parsimony, and high entropy value (0.993), indicating excellent classification accuracy. The smallest class in the two-class model comprised 36.8% of the total sample, meeting the minimum threshold for a meaningful subgroup.

Learners in Profile 1 (*n* = 99, 36.8%) demonstrated moderate levels of enjoyment across all FLE indicators, with item means ranging from approximately 3.47 to 3.89. In contrast, Profile 2 (*n* = 170, 63.2%) exhibited high levels of enjoyment, with means ranging from 4.45 to 4.99 across all indicators. Overall, the pattern reveals clear differentiation between the two groups in terms of both emotional and cognitive enjoyment dimensions.

A one-way ANOVA was conducted to examine whether learners with different FLE profiles differed in their classroom engagement (FCE). Results revealed a significant effect of FLE profile on LCE, *F*(1, 267) = 105.39, *p* < 0.001, η^2^ = 0.28, indicating a large effect size. Learners in the high-FLE profile (M = 4.51, SD = 0.65) reported significantly higher classroom engagement than those in the low-FLE profile (M = 3.68, SD = 0.62).

Taken together, the LPA and ANOVA results indicate that learners can be categorized into two distinct enjoyment profiles, which significantly differ in their classroom engagement. These findings complement and enrich the SEM results by revealing a two-profile solution that substantiates the mediating role of FLE between AIL and FCE in AI-enhanced language learning contexts.

## 5 Discussion

### 5.1 The predictive and mediating mechanism among AIL, FLE, and FCE (RQ1)

The first research question examined how AI literacy (AIL) influenced learners' foreign language enjoyment (FLE) and classroom engagement (FCE), and whether FLE mediated this relationship. The results of the structural equation model revealed that AIL had a significant positive effect on FLE, and FLE significantly predicted FCE. However, the direct effect of AIL on FCE was not significant, indicating that FLE fully mediated the relationship between AIL and FCE. This pattern suggests that AI literacy does not translate into engagement by itself, but operates by reshaping learners' emotional experiences of AI-mediated language learning.

The non-significant negative direct effect of AIL on FCE after FLE was included in the model may appear to suggest a suppression effect. However, additional multicollinearity diagnostics indicated that this explanation is unlikely. Specifically, the variance inflation factor (VIF = 1.769) and tolerance value (0.565) were both within recommended thresholds, suggesting that multicollinearity was not a serious concern. Instead, the findings indicate that the positive association between AI literacy and classroom engagement is primarily transmitted through learners' emotional experiences rather than through a direct pathway.

Although AIL and FLE were moderately correlated, they remain conceptually distinct constructs. AI literacy reflects learners' knowledge, skills, and confidence in using AI technologies for learning, whereas foreign language enjoyment represents learners' positive emotional experiences during language learning. The moderate association between these constructs is theoretically expected, as learners with higher AI literacy are more likely to perceive AI-supported learning activities as manageable, useful, and rewarding, thereby experiencing greater enjoyment. Nevertheless, the two constructs capture different aspects of AI-enhanced language learning and should not be regarded as conceptually redundant.

This finding highlights the essential role of emotional experience in AI-enhanced language learning. Rather than directly increasing students' behavioral engagement, AI literacy appears to foster learners' engagement indirectly by promoting positive emotions such as enjoyment and satisfaction. This result aligns with recent longitudinal evidence from AI-mediated L2 education, which shows that both enjoyment and engagement increase over time (Zhao, 2025). Through developing AI literacy, students gain greater control over their learning process, feel more capable of handling AI-supported tasks, and experience a sense of success when effectively using AI tools—all of which contribute to heightened enjoyment.

The magnitude of the standardized effects further supports this interpretation. The substantial standardized indirect effect (β = 0.661) suggests that the association between AI literacy and classroom engagement is largely accounted for by foreign language enjoyment, whereas the negligible standardized direct effect (β = −0.068) indicates little independent association between AI literacy and classroom engagement after accounting for learners' emotional experiences. Although FLE was strongly correlated with FCE (r = 0.77), the two constructs are conceptually distinct. FLE reflects learners' positive emotional experiences, whereas emotional engagement represents one dimension of classroom engagement. Therefore, the observed correlation reflects their theoretical relatedness rather than conceptual overlap.

The mediation effect of FLE is also consistent with both Control-Value Theory (Pekrun, 2006; Davis et al., 1989). From a CVT perspective, AI literacy may affect learners' perceived control over AI-assisted tasks and increases the value they assign to the learning activities, thereby eliciting positive academic emotions such as enjoyment. At the same time, TAM suggests that higher AI literacy strengthens learners' perceptions of the usefulness and ease of use of AI tools, which contributes to more positive affective responses and greater willingness to engage. These findings reposition AI literacy not merely as a technological competence, but as an affective catalyst that reshapes learners' emotional engagement with AI-mediated instruction.

This finding further complements previous research showing that enjoyment acts as a strong emotional driver of engagement (Dewaele and MacIntyre, 2014). In the context of AI-supported instruction, learners who possess higher levels of AI literacy are more likely to appreciate the learning process, perceive AI as a supportive partner rather than a threat, and thus become more actively involved in classroom activities. Therefore, enjoyment functions as a bridge linking learners' digital competence and their behavioral involvement, suggesting that affective experience is a key mechanism in AI-mediated language learning environments.

### 5.2 Learner profiles based on enjoyment patterns (RQ2)

The second research question was designed to identify distinct learner profiles characterized by variations in foreign language enjoyment (FLE). Latent profile analysis (LPA) was conducted using three indicators of FLE, and several statistical criteria (AIC, BIC, adjusted BIC, entropy, LMR-A, and BLRT) were considered to determine the optimal number of profiles. Among the competing models (two-, three-, and four-profile solutions), the two-profile model demonstrated the best balance between model fit and interpretability. Although the BIC value continued to decrease slightly as the number of profiles increased, the reduction was marginal, and the entropy value dropped in the three- and four-profile solutions, indicating poorer classification accuracy. Moreover, the LMR-A and BLRT tests were significant for the two-profile model but non-significant thereafter, further confirming that the two-profile solution was statistically optimal.

The two identified profiles were labeled as low-FLE learners (*n* = 99, 36.8%) and high-FLE learners (*n* = 170, 63.2%). Learners in the high-FLE profile reported consistently higher mean scores across all FLE indicators, suggesting stronger enjoyment in classroom activities, peer interaction, and AI-assisted learning tasks. In contrast, those in the low-FLE profile displayed lower levels of enjoyment and less positive affect toward language learning and AI-supported practices.

These profiles primarily reflected differences in the overall level of FLE rather than qualitatively distinct response patterns. Although the identified profiles were largely level-based, they nevertheless indicate that learners vary substantially in the intensity of their enjoyment in AI-enhanced language learning. This finding is broadly consistent with previous person-centered studies reporting differences in learners' motivational and emotional experiences (Zhang et al., 2026; Yoon and Ahn, 2025). However, the absence of shape differences suggests that the identified profiles should be interpreted as quantitative rather than qualitative distinctions.

Although the identified profiles mainly differed in overall enjoyment levels, they provided a useful basis for subsequent comparisons of classroom engagement. As the ANOVA results (RQ3) indicate, learners with higher FLE also reported significantly higher classroom engagement, suggesting that variations in enjoyment intensity are associated with differences in behavioral participation.

### 5.3 Differences in engagement and AI literacy across FLE profiles (RQ3)

The third research question explored whether there were significant differences in classroom engagement (FCE) across the identified FLE profiles. The ANOVA results showed that the variance of FCE between groups was homogeneous (Levene's test: *p* = 0.582), and there was a statistically significant difference in FCE between the two FLE profiles [*F*(1, 267) = 105.39, *p* < 0.001, η^2^ = 0.28]. Learners in the high-FLE profile reported significantly higher levels of engagement than those in the low-FLE profile.

These findings provide additional empirical support for the SEM results, confirming that enjoyment plays a crucial role in shaping students' engagement in AI-integrated learning contexts. As proposed by Control**–**Value Theory (Pekrun, 2006), positive emotions—enjoyment included—tend to arise when learners perceive themselves as having high control over a task and attribute substantial value to it. When these appraisals are strong, learners tend to demonstrate greater curiosity, persistence, and willingness to invest effort in classroom activities, resulting in sustained behavioral engagement.

Furthermore, this result is consistent with studies emphasizing the affective basis of engagement (Reschly and Christenson, 2012; Mercer and Dörnyei, 2020). Enjoyment, as an affective variable, fosters a sense of connectedness and agency in learning, which is particularly important in AI-mediated contexts where learners interact not only with peers and teachers but also with intelligent systems. The significant difference in engagement between the high- and low-FLE groups substantiates the notion that affective experience is the driving force behind behavioral and cognitive involvement. From a theoretical perspective, these profiles reflect qualitatively different control**–**value appraisals toward AI-mediated tasks, suggesting that AI-enhanced classrooms generate distinct emotional pathways rather than uniform affective responses.

Taken together, the ANOVA results corroborate the mediation pattern found in the SEM model. Learners with higher enjoyment levels, strengthened by their AI literacy, tend to exhibit greater classroom engagement. This convergence of findings across analytical approaches underscores that emotional experience functions as a bridge connecting technological literacy with active learning participation.

## 6 Conclusions and limitations

This study examined the relationships among AI literacy (AIL), foreign language enjoyment (FLE), and classroom engagement (FCE) in AI-enhanced English learning contexts using both variable-centered and person-centered approaches. The findings offer convergent evidence that learners' emotional experiences play a central role in translating AI literacy into active classroom engagement.

Structural equation modeling (SEM) revealed that AIL significantly predicted FLE, while its effect on FCE was fully mediated by FLE. The non-significant direct path from AIL to FCE suggests that AI-related competence alone does not automatically have a strong association with higher engagement; rather, enjoyment functions as a key emotional mechanism through which AI literacy provokes learners' behavioral, cognitive, and emotional involvement in learning.

Complementing these findings, latent profile analysis (LPA) identified two distinct learner profiles based on levels of FLE: a high-FLE group and a low-FLE group. Learners in the high-FLE profile reported consistently stronger positive affect and social enjoyment in AI-supported learning, whereas those in the low-FLE profile exhibited comparatively weaker emotional responses. One-way ANOVA further demonstrated that learners with high FLE showed significantly higher levels of classroom engagement than their low-FLE counterparts, with a large effect size. Together, these results provide robust support for the mediating role of enjoyment and highlight meaningful individual differences in affective engagement with AI-enhanced language learning.

Overall, this study contributes to the growing literature on AI-enhanced language education by demonstrating that the pedagogical value of AI literacy is realized primarily through learners' emotional experiences rather than through direct behavioral effects. The findings underscore the importance of considering affective processes and learner profiles when examining the impact of AI-related competencies on learning engagement.

The findings also yield important pedagogical implications. First, the full mediation of FLE suggests that emotionally supportive instructional design should be integral to AI-assisted language teaching. Beyond developing learners' technical skills, educators should also foster positive emotional experiences, such as enjoyment, curiosity, and a sense of accomplishment, when integrating AI tools into learning activities. Second, the identification of distinct FLE profiles highlights the need for differentiated support in AI-mediated classrooms. Learners with lower enjoyment may benefit from additional scaffolding, autonomy-supportive feedback, or guided interaction with AI tools, whereas high-FLE learners can be encouraged to engage in more challenging or creative AI-based tasks. Finally, embedding AI literacy instruction within meaningful and emotionally engaging learning contexts may help maximize both cognitive and affective learning outcomes.

Several limitations should be acknowledged. First, the sample was drawn from a single university and consisted of students enrolled in an AI-enhanced language learning course. While this context-specific sampling might boost ecological validity within the target instructional setting, it may limit the generalizability of the findings to other institutional or instructional contexts.

Second, the sample size (N = 269), although adequate for SEM-based analyses, is relatively constrained for the combined use of SEM and latent profile analysis (LPA). In particular, LPA typically requires larger samples to ensure the stability and replicability of identified profiles. Future research with larger and more diverse samples is encouraged to validate and potentially extend the current profile structure.

Third, although the structural equation model demonstrated acceptable CFI, TLI, and SRMR values, the RMSEA and χ^**2**^/df exceeded commonly recommended thresholds, indicating that the overall model fit was not optimal. Inspection of the modification indices suggested that improving model fit would primarily require data-driven cross-loadings and correlated residuals that lacked sufficient theoretical justification. Therefore, the theoretically specified model was retained, and the findings should be interpreted with appropriate caution. Future research is encouraged to further refine the measurement and structural models and examine the relationships among the constructs in different samples.

In addition, the two latent profiles identified in this study mainly reflected quantitative differences in the level of foreign language enjoyment rather than qualitatively distinct profile shapes. Therefore, the incremental contribution of the person-centered approach should be interpreted with caution. Future studies using larger and more heterogeneous samples may identify more differentiated learner profiles.

Fifth, the use of cross-sectional data restricts causal interpretation; therefore, the findings should be interpreted as associative rather than causal. Future studies employing longitudinal or experimental designs are needed to establish the temporal and causal relationships among AI literacy, foreign language enjoyment, and foreign classroom engagement.

Finally, the inclusion of qualitative methods in future research could provide deeper insights into how learners experience and negotiate emotion and engagement in AI-enhanced language learning.

## Data Availability

The datasets presented in this article are not readily available because data will be made available on request. Requests to access the datasets should be directed to dj3253@163.com.
